# Effectiveness of the Addition of a Cross-Education, Mirror Therapy, and Virtual Reality Exercises in Patients with Anterior Cruciate Ligament Reconstruction: The CROSSMIRV Trial Protocol

**DOI:** 10.3390/jcm15145552

**Published:** 2026-07-15

**Authors:** Felipe Araya-Quintanilla, Héctor Gutiérrez-Espinoza, Joaquín Salazar-Méndez, Sebastián Pinto-Concha, Francisca Ruiz-Riquelme, José Francisco López-Gil

**Affiliations:** 1 Escuela de Kinesiología, Facultad de Ciencias de la Rehabilitación y Calidad de Vida, Universidad San Sebastián, Santiago 7510602, Chile; felipe.arayaq@uss.cl; 2 Faculty of Education, Universidad Autónoma de Chile, Santiago 7500912, Chile; hector.gutierrez@uautonoma.cl; 3 Escuela de Ciencias del Deporte y Actividad Física, Facultad de Salud, Universidad Santo Tomás, Talca 3480094, Chile; jsalazar13@santotomas.cl; 4 Servicio de Medicina Física y Rehabilitación, Centro Las Hualtatas, Santiago 7650710, Chile; kinepinto@gmail.com; 5 Servicio de Medicina Física y Rehabilitación, Clínica Indisa, Santiago 7520440, Chile; fruizriquelme@gmail.com; 6 School of Medicine, Universidad Espíritu Santo, Samborondón 092301, Ecuador; 7 Vicerrectoría de Investigación y Postgrado, Universidad de Los Lagos, Osorno 5290000, Chile

**Keywords:** anterior cruciate ligament reconstruction, exercise therapy, mirror movement therapy, virtual reality, randomized controlled trial

## Abstract

**Background**: Based on the available evidence, there are no published clinical trials that have analyzed the effectiveness of adding cross-education, mirror therapy, and virtual reality exercises (combined) to standard treatment to improve clinical outcomes in patients after anterior cruciate ligament (ACL) reconstruction. **Methods**: A total of 58 patients with ACL reconstruction, aged 18–50 years, will be randomized (1:1) to two treatment arms. The control group (n = 29) will receive a standard physiotherapy program based on a consensus developed by the Sport and Health Rehabilitation Guidelines. The experimental group (n = 29) will receive a novel physiotherapy program that includes cross-education exercise with visual biofeedback, mirror therapy, and virtual reality in addition to the standard exercise program. Five evaluations will be performed: at the beginning of physiotherapy treatment (baseline), at 6 weeks, 12 weeks, 24 weeks and 52 weeks after surgery. The primary outcome will be passive knee extension range of motion at 6 weeks (for sample size), assessed by goniometry; the remaining outcomes are secondary and exploratory. The secondary outcomes measures will be pain intensity, fear of movement, the level of anxiety, knee function, and isometric quadriceps strength. **Discussion**: We hypothesize that patients who receive a novel physiotherapy program that includes cross-education with visual biofeedback, mirror therapy, and virtual reality will show greater benefits in knee extension and other clinical outcomes than those who receive a standard physiotherapy program. **Conclusions**: The trial proposed in this protocol will provide new data on the effectiveness of the combination of cross-education, motor imagery, and virtual reality therapy added to a standard physiotherapy program compared with the standard physiotherapy program alone in patients who undergo ACL reconstruction. The findings will generate new insights into the effects of the interventions and expand the multimodal rehabilitation strategies available to patients who undergo this surgery.

## 1. Introduction

Anterior cruciate ligament (ACL) injury is a health problem that generates significant costs in a person’s years of productive life. In the United States, approximately 200,000 ACL injuries occur each year, and an estimated 400,000 ACL reconstructions are now performed annually and generating expenses to the health system between US$625 million and US$1 billion annually [1,2,3]. This volume reflects a sustained upward trend: over a 17-year period, the age-standardized incidence of primary ACL reconstruction rose by roughly 26% (from 40.6 to 51.2 per 100,000 people aged ≥10 years), with the increase being most pronounced among women [3]. Based on epidemiological studies, after 2 years of follow-up, there is a high risk of re-tear after ACL surgery. This risk increases exponentially to 25 times more when physiotherapeutic treatment fails in the early stages [4,5]. Evidence shows that these injury risks after ACL reconstruction are because patients who undergo surgery show quadriceps weakness, abnormal movement patterns, and low neuromuscular activity [6,7]. Moreover, in early stages, a lack of knee extension is the most prevalent deficit in this clinical condition, which has been associated with poor knee function and low long-term quadriceps muscle strength [8]. Likewise, other studies have shown that persistent quadriceps weakness and a lack of knee extension contributes to knee instability, disability, risk of knee osteoarthritis, and increased re-injury rates at 2 years after surgery [9,10].

Previous evidence has attempted to explain how this clinical impairment could generate neurophysiological maladaptations after ACL injury [11,12]. Kapreli et al. [11]. demonstrated that patients with ACL injury have low activation in sensorimotor cortical areas assessed with functional magnetic resonance. Diekfuss et al. [12] showed that patients with ACL reconstruction have a stronger functional connection between a cortical sensory-motor region and a cerebellar region responsible for balance and coordination in the lower limb. Other studies have demonstrated that patients with ACL reconstruction show an increased motor threshold and low excitability of the motor cortex, generating a deficient “output” of descending motor neurons toward the muscles of the lower extremity [13,14,15,16]. Therefore, patients with ACL tears may have a greater motor planning demand to generate optimal movement and better function of the lower extremity. Additionally, other clinical changes are related to a greater fear of movement of re-injury and pain intensity during ACL reconstruction rehabilitation [17]. In the same way, these clinical manifestations—including fear of movement and anxiety associated with aspects related to pain—are caused by the increased neural activity of mesolimbic areas and the amygdala [11,17].

The current approach of physiotherapy after ACL surgery focuses on improving the functional impairment of the clinical condition and preventing a re-injury: improving early range of motion (ROM), increasing the quadriceps muscle strength, and achieving a good functional gait and knee function [18]. However, studies that have analyzed the effect of conventional physiotherapy protocols have reported a lack of knee extension, decreased activation of the quadriceps, gait asymmetry, and a greater probability of re-tear up to 2 years after reconstruction surgery [19,20]. Therefore, the appropriate early physiotherapy program for patients who undergo ACL surgery remains a topic of debate in the literature. There is recent evidence that graded motor imagery (GMI), cross-education exercises, and virtual reality are therapeutic tools that attempt to facilitate neuromuscular activation as well as sensory reorganization of the cerebral cortex, and to improve functional outcomes [21,22]. 

GMI includes three stages: recognizing judgments of laterality, movement imagery, and mirror therapy. Mirror therapy effectively improves motor cortex activation and decreases the neural activity of pain-processing areas [23]. Additionally, mirror therapy activates the premotor cortex and supplementary motor area to increase motor neuron function to the peripheral muscle [24]. Cross-education refers to strength gain observed in the contralateral limb after a unilateral training program [25]. A meta-analysis reported that quadriceps muscle strength can be increased by up to 18% by working the contralateral limb [26]. Moreover, applying motor activation and visual strategies for the limb contralateral to the affected limb improves information processing at the cortical level, reducing pain perception and increasing contralateral muscle strength [25,27]. Finally, another systematic review, conducted in patients with knee osteoarthritis, specifically indicated that virtual reality improves functional outcomes of the lower limb [28]. Therefore, implementation of these strategies from the early stages of a standard physiotherapy program may ameliorate changes in cortical reorganization and thus improve muscle activation and prevent a re-tear in these patients. Recent evidence supports the use of cross-education in this population: in a randomized controlled trial, adding contralateral cross-education training to conventional rehabilitation after ACL reconstruction produced greater improvements in knee function, kinematic parameters, dynamic balance, and plantar pressure than conventional rehabilitation alone [29]. However, that trial evaluated cross-education in isolation, and did not combine it with visual biofeedback, mirror therapy, or virtual reality. Therefore, to our knowledge, no published clinical trial has analyzed the effectiveness of adding cross-education alongside visual biofeedback, mirror therapy, and virtual reality exercises to standard treatment. We hypothesize that, compared with standard physiotherapy alone, patients allocated to the novel physiotherapy program (multimodal intervention) rather than to standard treatment will show a significantly greater improvement in passive knee extension range of motion at the 6-week primary endpoint. Finally, the aim of this study is to describe the rationale and methods of a randomized clinical trial that will assess the effectiveness of a multimodal intervention that includes cross-education alongside visual biofeedback, mirror therapy, and virtual reality exercises added to standard treatment to improve clinical outcomes in patients who undergo ACL surgery.

## 2. Materials and Methods

### 2.1. Study Design/Setting

**Figure 1:**
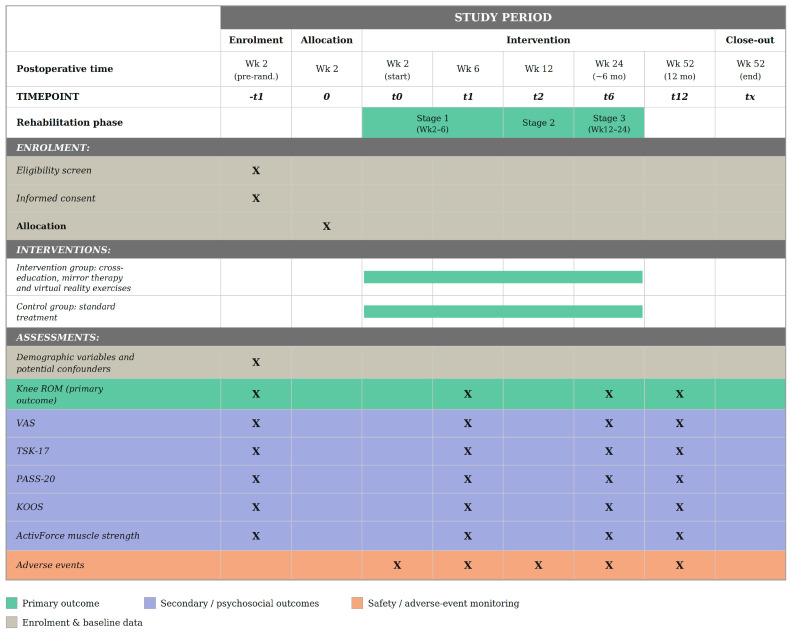
SPIRIT schedule of enrolment, interventions, and assessments. Columns indicate the corresponding postoperative time for each timepoint (Week 2 at enrolment/allocation, Week 6, Week 12, Week 24 [~6 months], and Week 52 [12 months]); the shaded band denotes rehabilitation Stages 1–3 (Section 2.3); row colors distinguish the primary outcome (knee ROM), secondary/psychosocial outcomes, safety/adverse event monitoring, and enrolment/baseline data, as detailed in the legend beneath the table.

This will be a single-blind, two-arm, parallel-group superiority randomized controlled trial, designed to assess the effectiveness of the addition of a novel multimodal physiotherapy program to standard treatment, which is superior to standard treatment alone for passive knee extension in patients undergoing ACL reconstruction. It will be conducted at the Physical Therapy Department of the Chilean Medical Center. This protocol is based on Standard Protocol Items: Recommendations for Interventional Trials (SPIRIT) guidelines, and the checklist is available in the Supplementary Materials [30]. The participants are residents who will mainly be recruited in the city of Santiago. The participants will be informed about the research, procedures, risks, and benefits by FA-Q. If they agree, then they will sign an informed consent form. Only those participants who read and agree to the protocol and who sign the informed consent form will be part of the study, following the schedule described in Figure 1.

### 2.2. Participants

This study will include 58 patients of the Private Clinical of Chile who undergo ACL reconstruction (patellar bone–tendon–bone, gracilis semitendinosus tendon, and/or allograft). All patients will be treated with postoperative relative rest for 4 days and will be prescribed an oral nonsteroidal anti-inflammatory drug twice a day for 14 days depending on the context of each patient. The patient will be referred to the Physical Therapy Department 2 weeks after surgery.

#### 2.2.1. Inclusion Criteria

To participate in this study, the participants will have to meet the following inclusion criteria: (1) men and women aged 18–50 years with an ACL reconstruction, who have undergone an independent reconstruction surgery of the ACL (with or without other injuries such as a meniscus tear with a meniscal suture, collateral ligament injury, posterior ligament sprain, etc.) with a normal body mass index (18.50–24.99 kg/m^2^); (2) agreement to participate in the trial and signing the informed consent form; and (3) the ability to follow simple orders in Spanish (based on a Mini-Mental State Test score >26 points).

#### 2.2.2. Exclusion Criteria

Patients will be excluded if they meet any of the following criteria: (1) unable to walk after surgery; (2) other pathologies associated with the knee joint including knee osteoarthritis, degenerative injuries, or patellar tendinopathy prior to surgery, posterior cruciate ligament injury, and re-tear of the ACL; (3) orthotic devices in the knee joint; and (4) some degree of cognitive impairment.

### 2.3. Interventions

During the trial, all participants will receive the same standardized postoperative regimen (relative rest for four days and oral non-steroidal anti-inflammatory drugs twice daily for 14 days), with paracetamol permitted as rescue analgesia. The following co-interventions will not be permitted during the supervised treatment period: additional supervised physiotherapy outside the trial, intra-articular or peri-articular injections, additional structured strengthening or exercise programs, and routine prophylactic bracing beyond that prescribed by the operating surgeon. Any analgesic use, unsupervised exercise, bracing, or additional treatment will be recorded at each assessment in the eCRF and incorporated as a covariate in sensitivity analyses.

The control group (n = 29) will receive a standard physiotherapy program based on the consensus statement of the Sport and Health Rehabilitation Guidelines MOON Group [31]. This program proposes to control signs and symptoms for 2 weeks after surgery. The main components at this stage are to control edema, to reduce pain, to achieve a normal gait pattern, and to start muscle activation of the quadriceps; and to provide the participant with education about their clinical condition and evolution. In stage 1 (0–6 weeks), local cryotherapy will be performed for 15 min in addition to conventional transcutaneous electrical nerve stimulation (TENS at 120 Hz and 50 Us).

From 0 to 2 weeks, four exercises will be performed: (1) Basic therapeutic mobilization of knee extension in the prone position, knee flexion in the supine position, and patellar mobilization. (2) Isometric exercise of the quadriceps in the supine position with 10 s of isometry for 30 repetitions. (3) Hamstring exercise in the sitting position with the knee slightly in flexion (the participant will be asked to push their foot toward the floor for the contraction). (4) Hip abduction exercises in the side position. The participant will be asked to take off one knee from the other and maintain the position for 5 s for 30 repetitions.

From 2 to 6 weeks, six exercises will be performed: (1) Basic therapeutic mobilization of knee extension in the prone position. (2) Knee flexion in the supine position with the feet supported against a wall to increase the ROM. (3) Semi-squat with the back supported on the wall, with three series of 15 repetitions. (4) Step-up on both legs, with 20 repetitions per leg. (5) Bridge of one leg in extension, maintaining the position for 10 s. (6) Hamstring with a red elastic band, with three series of 20 repetitions. During this phase, aerobic exercise will be added (20 min on a cycle ergometer). Stage 2 (6–12 weeks) will involve complete knee ROM, dynamic knee exercises, painless control, small squats without pain and the “giving way” feeling, and knee flexion at 8 degrees without pain. Seven exercises will be performed: (1) Full squats in the protected range, with three series of 20 repetitions. (2) Leg press between 90 and 0 degrees according to a tolerable load, with three series of 12 repetitions. (3) Hamstring curl in press, with three series of 20 repetitions. At this stage, four dynamic control and balance exercises will be added. (4) Unipodal disturbance training. (5) Diagonal landslides on stable surfaces. (6) Jumps without pain on unstable surfaces. (7) Ball launch exercises to the wall with unstable surfaces, with the addition of a cognitive task, where there are three series of 20 repetitions. This phase will also include aerobic exercise (40 min on a cycle ergometer).

The specific components of stage 3 (12–24 weeks) include jumping without pain, and achieving daily and sports life activities without a sense of fear of movement or injury. The same exercises described in stage 2 will be performed, increasing the progressions and load (to 2 kg) according to the tolerance of each participant. In addition, four exercises will be added.

(1) Management and deceleration changes exercises with obstacles for 5 min.
(2) Squats at 90 degrees, maintaining the position for 10 s for 30 repetitions.
(3) Exercises on an unstable platform with cognitive tasks in a unipodal position.
(4) Exercises for reintegration to sport activity according to the participant’s preference.

This phase will also incorporate aerobic exercise (50 min on a cycle ergometer). All sessions in stage 3 will last approximately 90 min. There will be three weekly sessions for 24 weeks.

**Figure 2:**
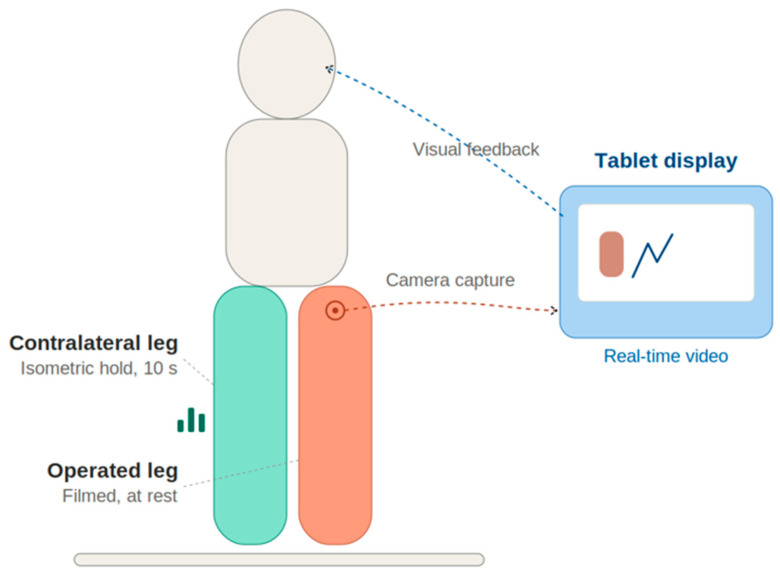
Schematic of the cross-education exercise with visual biofeedback. The participant watches a real-time video of the operated limb on a tablet screen (camera capture, dashed red arrow) while simultaneously performing an isometric contraction of the contralateral limb (green, 10 s hold); the visual feedback loop (dashed blue arrow) reinforces the cross-education effect. Orange: operated limb, filmed at rest.

The intervention group (n = 29) will receive a novel physiotherapy program that includes cross-education exercise with visual biofeedback, mirror therapy, and virtual reality in addition to the standard treatment based on the MOON group guidelines (as described above for the control group). This intervention also includes three stages. In stage 1 (0–6 weeks), cross-education exercises will be added to the first two weeks. The specific cross-education components are strength gain of the injured limb, training the contralateral side, increasing the excitability of corticospinal pathways, and reducing the inhibition of the motor cortex due to the injury. To perform cross-education with visual biofeedback, while in the supine position, the participant will be shown a video of an isometric contraction of quadriceps in real time on an electronic device (tablet). The participant will be asked to look at the video during each contraction and simultaneously perform an isometric contraction of the contralateral (non-operated) leg for 10 s, with 30 s of rest. This exercise will be performed for 15 min (Figure 2).

**Figure 3:**
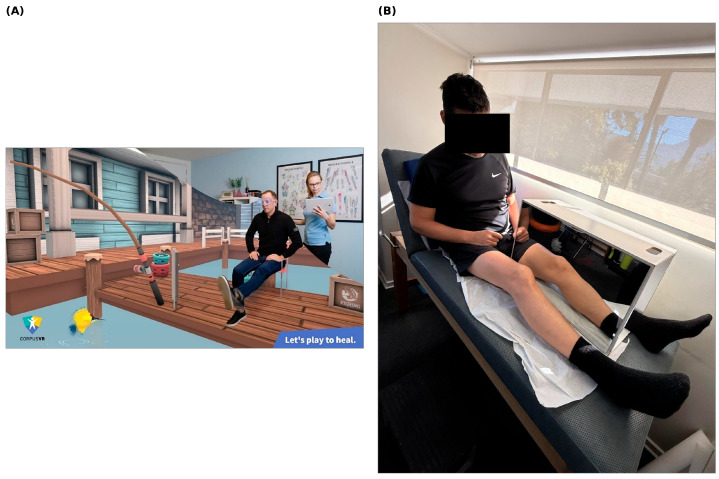
Components of the multimodal rehabilitation intervention. (**A**) Virtual reality lower-limb exercise (Corpus VR—InMotionVR application, or native application on Oculus Meta Quest 3S; software version 6.2). Objective: sustained active knee extension of the operated limb to “catch” virtual objects (fishing game) within a target range of motion. Progression criteria: increasing target distance/range of motion, session duration progressing from 15 to 30 min, and increasing task complexity/speed as tolerated. (**B**) Mirror therapy with cross-education of the operated lower limb (parasagittal mirror positioned in the midline between the limbs, reflective surface facing the non-operated side so that its reflection is superimposed over the operated limb; mirror/apparatus model to be specified). Objective: repeated active knee flexion–extension of the non-operated limb while the participant observes its mirror reflection in place of the operated limb, to elicit cross-education and sensorimotor engagement of the operated side. Progression criteria: increasing repetitions and sets, range of motion, and movement speed, with session duration progressing from 15 to 30 min and increasing task complexity as tolerated (to be specified). Written informed consent for publication of this image was obtained from the participant; facial features have been masked to preserve anonymity.

In stage 2 (2–6 weeks), the same exercises mentioned in stage 1 will be performed, increasing the progressions of isometry to 20 s, with 45 s of rest. In addition, mirror therapy exercises will be added. While the participant is sitting, a mirror will be installed between both limbs. The participant will be asked to perform knee-extension movements with the non-operated limb, which should simulate the injured limb. The participant will be asked to perform the movement for 10 min. These interventions will be performed at least two times a week. The specific components of mirror therapy are a decrease in the somatosensorial areas related to pain and activation of brain motor areas to increase neuromuscular activity without generating movement of the limb to avoid pain and muscle fatigue. In addition, during the last two weeks, virtual reality exercise will be performed using a lower-limb rehabilitation application (Corpus VR—InMotionVR, version to be specified at the time of publication, or the native application running on Oculus Meta Quest 3S). While seated, the participant will wear the virtual reality headset and perform a lower-limb virtual reality game consisting of fishing for different objects from a virtual lake (Figure 3), using active knee extension of the operated limb to reach and “catch” each target. The objective of the game is to sustain controlled, repeated active knee extension within a progressively increasing range of motion while maintaining task engagement through the gamified format. Progression will be criterion-based: as the participant demonstrates adequate control and a pain-free range of motion, the target distance and range of motion will be increased, session duration will progress from 15 to 30 min, and task complexity/speed will be increased as tolerated. This exercise will be performed for 30 min, twice per week. The specific components of virtual reality are an increase in neural activity in the motor and premotor cortices to improve connectivity in corticomotor pathways and to improve muscle activation. All sessions will last approximately 60 min. Cross-education exercises with visual biofeedback, mirror therapy, and virtual reality will be added to the standard therapy in each session for each participant. There will be three weekly sessions for 24 weeks.

Exercise progression will be based on each participant’s ability. A maximum score of 4/10 on the visual analog scale (VAS), a 6/10 on the Borg scale, and correct technique, as determined by the physical therapist, will be considered for progressing to the next level. The participants will be instructed that if pain and/or inflammation increases and does not subside until the next session, then they should attend their session as normal. Signs and symptoms will be managed, and exercises will be adapted according to tolerance and the corresponding stage. Both groups will receive individualized, in-person, supervised treatment in the clinic’s physical therapy department. Physical therapists in both groups (with more than 10 years of experience in musculoskeletal areas) will be trained in the respective intervention to maintain program fidelity and will be provided with a printed guide containing the complete treatment content. Adherence will be defined as a priori as attendance at ≥ 80% of the 72 scheduled sessions (three sessions per week for 24 weeks). Missed sessions will be rescheduled within the same week wherever possible; a participant who misses two consecutive sessions will be contacted by telephone to identify barriers and encourage retention. Worsening symptoms or a suspected adverse event will trigger immediate clinical assessment. The allocated intervention will be discontinued in the event of graft re-tear, surgical revision, a serious adverse event, a clinical decision by the treating orthopedic surgeon, or withdrawal of consent. Participants who discontinue the allocated intervention will nonetheless be invited to complete all scheduled outcome assessments at 6 weeks, 6 months, and 12 months, in accordance with the intention-to-treat principle. Adherence, reasons for missed sessions, and reasons for discontinuation will be recorded in the electronic case report form (eCRF). Treatment fidelity will be monitored throughout the trial and is conceptually distinct from participant adherence, which is defined as attendance at a minimum of 80% of the 72 scheduled sessions; whereas adherence is a participant-level construct, fidelity concerns whether the intervention is delivered, received, and enacted as intended. Fidelity will be addressed across the five domains of the NIH consortium, At the level of design, both the control and experimental programs are fully manualized, specifying for every stage the exercises, sets, repetitions, progression criteria, equipment, and session frequency, so that the intended dose and content are pre-defined and identical across therapists. At the level of training, all treating physiotherapists, each with more than ten years of musculoskeletal experience, will complete standardized training on both protocols using the printed guide and will then undergo a competency check in which they must demonstrate correct delivery of each experimental component (cross-education with visual biofeedback, mirror therapy, and virtual reality) before treating any participant; booster training will be provided if drift is detected during the trial.

Delivery will be monitored session by session. For every session, the treating physiotherapist will complete a standardized checklist recording the exercises performed, the sets and repetitions, the progression decisions taken against the pre-specified criteria, the equipment used (Corpus VR InMotionVR/Oculus Meta Quest 3S), the session duration, and any protocol deviation together with its reason. In addition, a random sample of at least 15% of sessions in each arm will be directly observed, in person or by video recording, by an investigator not involved in delivering treatment and scored against a fidelity checklist; the proportion of key components delivered correctly will be summarized for each arm and reported. When fidelity falls below a pre-specified threshold of 80% of checklist items, corrective feedback and, where necessary, re-training will be provided. To confirm that the intervention is not only delivered but also received and enacted, participants’ understanding and correct performance of the prescribed exercises will be verified at each session, and completion of any home exercises will be captured in the eCRF. To minimize cross-contamination between arms, the experimental components—visual biofeedback, mirror, and virtual-reality equipment—will be used only during scheduled experimental-group sessions and will not be accessible to control participants; the two groups will be treated in separate rooms and, where scheduling permits, at separate times; control participants will not receive any of the experimental modalities; and participants will be asked not to seek additional physiotherapy or the experimental modalities outside the trial, with any co-interventions recorded in the eCRF and any suspected contamination documented and reported. All fidelity data, comprising the session checklists, observation scores, recorded deviations, and contamination reports, will be reviewed periodically by the principal researcher (FA-Q) as part of the trial’s internal monitoring, and a summary of fidelity, including the percentage of audited sessions and the mean component-delivery scores, will be reported alongside the trial results.

### 2.4. Outcome Measures

Participants will undergo five standardized assessments across the postoperative follow-up, scheduled at week 2 (baseline), week 6, week 12, week 24 (~6 months), and week 52 (12 months). The baseline assessment (week 2) will capture demographic variables and potential confounders together with the pre-intervention values of all efficacy outcomes, establishing the reference against which subsequent change is estimated. Outcomes are organized hierarchically. The single primary (confirmatory) outcome is the adjusted between-group difference in passive knee extension ROM at the 6-week postoperative assessment—the timepoint at which the intervention is hypothesized to exert its principal effect and the only outcome for which the trial is powered (Section 2.5). Passive knee extension ROM at the later assessments (24 weeks and 12 months) and all remaining efficacy outcomes—pain intensity (VAS), kinesiophobia (TSK-17), pain-related anxiety (PASS-20), knee function (KOOS), isometric quadriceps strength, and thigh circumference—are designated secondary and exploratory (hypothesis-generating). Re-tear and adverse events are safety outcomes. Two physiotherapists who are not part of the research team will perform the measurements. They will be blind to group assignments to avoid possible biases in the measurement.

#### 2.4.1. Primary Outcome Measure

Knee extension will be measured by the Baseline goniometer (precision: ±2°) for passive knee ROM. Passive knee extension ROM was selected as the primary outcome because a loss of terminal knee extension is the most prevalent early impairment after ACL reconstruction and is consistently associated with quadriceps weakness, gait asymmetry, poor knee function, an increased risk of knee osteoarthritis, and higher re-injury rates [8,9,10]. Extension will be measured in the supine position. The goniometer axis will be placed over the lateral epicondyle of the femur, the proximal arm will be placed on the lateral midline of the femur, and the distal arm will be placed on the lateral midline of the fibula. Each movement will be measured three times, and the average of these measurements will be used for the analysis. The goniometer has good intra-rater reliability (with an intraclass correlation coefficient of 0.90–0.99) when consistent body landmarks are used [32]. For ROM, the minimal clinically important difference (MCID) has not been established.

#### 2.4.2. Secondary Outcome Measures

Pain intensity will be assessed using the VAS, which consists of a horizontal line 10 cm in length, where the left end represents 0 or “painless” and the right end represents 10 or “worst pain imaginable.” The participant will be asked to mark with a vertical line the magnitude of the pain that they feel at the time of the evaluation. It is a one-dimensional, simple, and reproducible assessment method [33]. The MCID has not been established for the VAS. Fear of movement will be assessed with the original 17-item Tampa Scale of Kinesiophobia (TSK-17). Each item is scored on a 4-point Likert-type scale that ranges from strongly agree (1) to strongly disagree (4). The total score ranges from 17 to 68. A higher score indicates more fear of movement and/or (re-)injury [34]. The MCID has not been established for the TSK-17. The anxiety level associated with pain will be evaluated with the Pain Anxiety Symptoms Scale Short Form 20 (PASS-20) scale. It comprises 20 items that uses a 6-point Likert scale from 0 (never) to 5 (always). This instrument has four dimensions: cognitive anxiety, pain related fear, escape and avoidance, and physiological anxiety. The total score ranges from 0 to 100 points, with a higher score indicating a higher level of pain related to pain [35]. The MCID has not be established for the PASS-20.

Knee function will be evaluated with the Knee Injury and Osteoarthritis Outcome Score (KOOS) questionnaire. This instrument contains 42 items, including five subscales: symptoms, pain, daily life activities, sports/recreation, and quality of life. The maximum score is 100 points, which indicates greater functionality. This questionnaire has proven to have good validity and inter-rater reliability [36]. The MCID is 18.3 points [37]. Isometric muscle strength will be evaluated with the ActivForce digital dynamometer. Testing will be performed with the participant seated on a standardized testing chair, hip flexed to 90° and knee flexed to 60°, trunk upright and arms crossed over the chest. The pelvis and the distal thigh will be stabilized with inelastic straps to prevent compensatory hip and trunk movement, and the dynamometer will be positioned perpendicular to the tibia at a fixed point 5 cm proximal to the medial malleolus, recorded for each participant to ensure consistency across assessments. Three maximal “make” tests of 3–5 s (“push as hard as possible, push, push, push”) each will be performed with 30 s of rest, and the peak value will be used for analysis. This method has proven to have good validity and reliability [38]. For anthropometry, a measuring tape will be used to assess the thigh perimeter of the affected and healthy limbs before and after treatment. The circumference of the muscle will be evaluated with the tape measure located 15 cm above the upper edge of the patella. Previous studies have demonstrated the reproducibility of this method [39].

#### 2.4.3. Potential Confounders

Comorbidities that may affect graft consolidation, meniscus tear, and outcome measures—including hypercholesterolemia, diabetes, and tobacco and alcohol consumption—will be evaluated with a data collection notebook. The dominant side, time of surgery, and treatments received prior to surgery will be recorded (physiotherapy and use of drugs, such as nonsteroidal anti-inflammatory drugs, analgesics, and opioids). Weight, height, and body mass index (BMI) will be evaluated with a SECA 213 stadiometer. BMI will be calculated as the weight (in kilograms) divided by the height (in meters) squared. The education level will be recorded as primary, secondary, technical-higher, or university. The level of physical activity will be assessed by the Global Physical Activity Questionnaire (GPAQ). This instrument was originally designed by the World Health Organization to be administered by an interviewer to assess physical activity. The questionnaire comprises 16 items that quantify the participant’s physical activity level within a normal active week to estimate the total weekly volume of moderate-to-vigorous physical activity. It includes three domains: work, transportation, and recreational activities. This questionnaire has demonstrated consistent validity and reliability [40]. For the socio-economic status, the education level will be classified as primary education (functionally illiterate, without any studies, or those who have not completed primary education), middle education (primary education, high school/secondary education, or baccalaureate), and university education (college or PhD degree). Occupation will be categorized as heavy load, light load, and sick leave in the last month.

### 2.5. Sample Size Calculation

The sample size was estimated in G*Power v3.1 (a priori, two independent means, two-sided; α = 0.05; power = 0.80). The expected effect on knee extension was informed by Liu et al. [29] in a randomized controlled trial of contralateral cross-education in early post-operative ACL reconstruction that assessed knee range of motion by goniometry and reported a significantly greater improvement in the cross-education group (group × time interaction partial η^2^ = 0.21, *p* < 0.01; a between-group difference in ROM improvement of ≈4°). Rather than power the trial on the large effect implied by that study, we adopted a conservative target: with 24 analyzable participants per group, the trial has 80% power (two-sided α = 0.05) to detect a standardized between-group difference of d ≈ 0.83 (partial η^2^ ≈ 0.15), smaller than the effect reported by Liu et al. Allowing for 20% attrition, this yields 29 participants per group (58 total). As a sensitivity analysis, under the proposed sample size, the trial detects a between-group difference of d ≈ 0.75 (29 per group) to d ≈ 0.83 (≈24 analyzable per group), corresponding to approximately 2.2–3.3° of passive knee extension for a plausible standard deviation of 3–4°—a magnitude bracketing the ≥3° extension clinical deficit associated with poorer outcomes [41]. Because the primary analysis is a repeated-measures ANCOVA adjusting for the baseline outcome value, this *t*-test-based estimate is conservative relative to the planned analysis.

### 2.6. Recruitment

Potential participants will be identified and referred by the center orthopedic surgeons at the collaborating centers (Clínica INDISA and Centro Las Hualtatas, Santiago, Chile) by October 2026, and this investigation is expected to finish in November 2027. Eligible patients will be approached by the principal investigator (FA-Q) at their initial physiotherapy referral (approximately two weeks after surgery), provided with a written participant information sheet and a study flyer, screened against the eligibility criteria, and given the opportunity to ask questions before written informed consent is obtained. The written consent form will include information regarding the background and purpose of the study, the exercise program, the outcomes, and the expected benefits and possible drawbacks.

### 2.7. Randomization and Blinding

**Figure 4:**
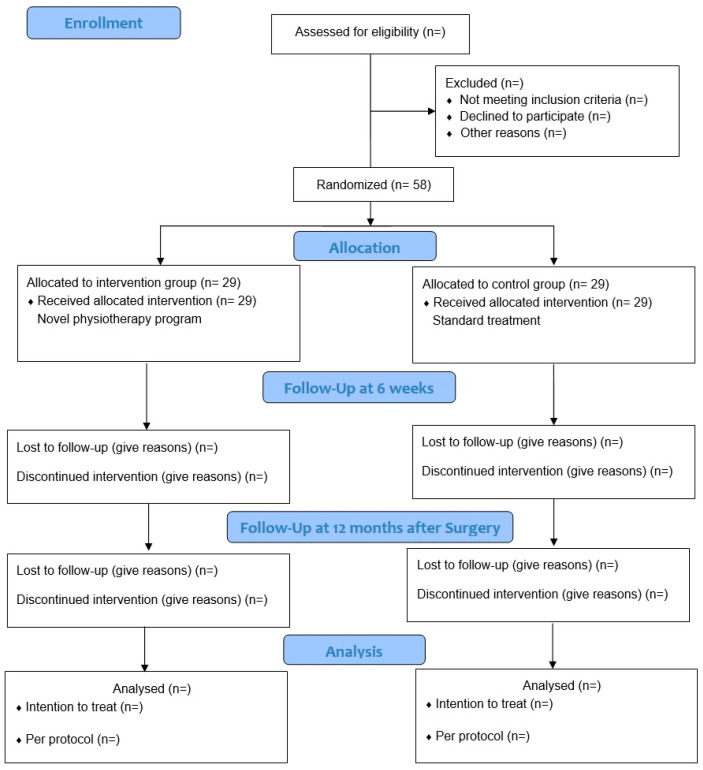
Anticipated participant flow through enrollment, allocation, follow-up, and analysis, in accordance with the CONSORT statement. As this is a trial protocol, the values shown for randomization and allocation (n = 58; n = 29 per group) reflect the planned target sample size; counts for screening, exclusions, and losses to follow-up are presented as anticipated categories to be completed once recruitment and follow-up are underway.

The participants will be randomly allocated to the two groups through a sequence of numbers generated by a computer program (1:1) before beginning the selection process. The group assigned to each participant will be kept in a sealed envelope so that only the researcher who makes the assignments will know to which group each participant belongs (Figure 4). Given the nature of the interventions, blinding physiotherapists and participants will not be possible. However, the assessors and statisticians will be blinded to which group the participants belong.

### 2.8. Availability and Management Data

Information obtained from the evaluation of each participant will be recorded on a paper print-out. The information will then be hand-written on paper or collected via the eCRF and entered in an Excel file for future statistical analyses. In accordance with the Personal Information Protection Act, the names of the participants will not be disclosed; they will be given a unique identifier number during the trial. The participants will be informed that the clinical data obtained in the trial will be stored on a computer and will be handled with confidentiality for a period of 5 years. The participants’ written consent will be stored by the principal researcher. The final de-identified trial dataset will be accessible to the principal investigator (FA-Q) and the trial statistician. No contractual agreements limit investigator access to the data. De-identified participant-level data and the statistical analysis code will be made available on reasonable request following publication.

### 2.9. Data Monitoring, Interim Analyses, and Auditing

Given the low risk, exercise-based nature of the interventions, the small single-center sample (n = 58), and the absence of any planned interim efficacy analyses, a formal independent Data Monitoring Committee was not established. Safety oversight is provided by the principal investigator (FA-Q), who reviews adverse-event logs and adherence rates after every 10 participants complete the 6-week primary-outcome assessment and who may consult the Scientific Ethics Committee of Clínica INDISA if any safety concern arises. No interim efficacy analyses are planned, and the trial will not be stopped early for efficacy. The principal investigator retains authority to suspend or terminate the trial for safety reasons—for example, if trial-related serious adverse events occur—in consultation with the Scientific Ethics Committee of Clínica INDISA. No independent external audit is planned; the principal investigator performs monthly internal monitoring of case-report-form completeness, adherence logs, and adverse-event records to verify protocol compliance, and these findings are documented and retained. As the trial is investigator-initiated with no external sponsor, this monitoring is internal to the investigator team.

Data collection and entry:. Outcome data will be recorded at each visit on standardized paper case report forms (source documents) by the blinded assessors. The trial database will be defined in EpiData Manager as a structured project file that fixes each variable’s type, permissible range, and internal-consistency (logic) rules; data will then be transcribed from the source documents into EpiData EntryClient by the trial data manager. To minimize transcription error, all primary-outcome fields, together with a pre-specified random 10% sample of every secondary-outcome field, will undergo independent double data entry into two separate files by two team members; the two files will be compared using EpiData’s duplicate-file validation, and every discordance will be reconciled against the original source document before database lock. Validation checks and error correction: At the point of entry, EpiData enforces the pre-specified range and logic checks, required-field rules, and legal-value (skip-pattern) constraints defined in the project file, so that out-of-range or inconsistent values are rejected or flagged as they are entered. Queries generated during entry or double-entry reconciliation will be resolved against the source documents. Every post-entry correction will be recorded in a dated, operator-attributed change log that preserves the original value, the corrected value, the reason for the change, and the identity of the person making it; source documents will never be over-written. Identifier-key management:A single master linkage log mapping participant names to the unique study identifier will be maintained by the principal investigator (FA-Q), stored separately from the trial dataset in an encrypted, password-protected file on the institutional server, and accessible only to the principal investigator and one named delegate. Only the study identifier will be entered into the EpiData database; no direct identifiers will be held in the analysis dataset. Access rights: Access will be role-based and documented in a delegation log: blinded assessors will record data on the paper source forms only; the data manager will hold entry, validation, and query-resolution rights to the EpiData database; the trial statistician will receive the locked, de-identified export for analysis; and the principal investigator will hold oversight access. Treating physiotherapists will have no access to the outcome database, preserving assessor blinding. Storage and security: The EpiData project and data files will reside on a password-protected, access-controlled institutional server with encryption at rest, automated daily backups, and restricted physical access; data will not be stored on portable or personal devices. Paper source documents and signed informed-consent forms will be held by the principal investigator in a locked cabinet within a restricted-access room, separately from the electronic data. Retention and destruction: In accordance with Good Clinical Practice and applicable Chilean personal-data protection legislation, de-identified trial data will be retained for a minimum of 5 years after publication, after which electronic files will be securely and irreversibly deleted and paper records cross-shredded. The identifier linkage log will be destroyed once data cleaning and the final analysis are complete, and no later than the end of the retention period. Data export: After database lock, the validated dataset will be exported directly from EpiData to SPSS for analysis; where a spreadsheet (Microsoft Excel) is used for any de-identified export, the file will be password-protected, stored solely on the secured institutional server, and version-controlled through sequentially dated, read-only file versions accompanied by the change log described above. No identifiers will be included in any export file.

## 3. Statistical Analysis

The continuous variables will be presented as the mean and standard deviation, and the categorical variables will be shown as the number and percentage. To determine whether parametric statistical tests are appropriate for use in data analysis, both statistical (Kolmogorov–Smirnov test) and graphical (Q-Q normal probability plot) methods will be used to assess whether the data follows a normal distribution.

Because the confirmatory hypothesis is tested through a single primary contrast (the adjusted between-group difference in passive knee extension ROM at 6 weeks), no adjustment for multiplicity is required for the primary analysis, and statistical significance for the primary outcome will be evaluated at a two-sided α = 0.05. All secondary and exploratory analyses—passive knee extension ROM at 24 weeks and 12 months and every secondary outcome—are exploratory and will not be subjected to formal multiplicity control; no secondary outcome is designated a key secondary endpoint for confirmatory testing, and no hierarchical or gatekeeping procedure is therefore applied. For these outcomes, we will report adjusted mean differences (or the corresponding effect measure) with 95% confidence intervals, interpreted as hypothesis-generating. We explicitly acknowledge that the family-wise type I error rate is not controlled across these comparisons, and we will refrain from confirmatory claims based on them. The primary estimator is the adjusted between-group difference in passive knee extension ROM at 6 weeks. This will be estimated by analysis of covariance (ANCOVA), with the 6-week passive knee extension value as the dependent variable, treatment group as the factor of interest, and baseline value of the outcome as a covariate, together with prespecified prognostic covariates; the treatment effect will be reported as an adjusted mean difference with its 95% confidence interval. The adjusted between-group difference at 6 weeks was chosen as the primary contrast in preference to a group-by-time interaction or a difference in change from baseline, because the primary endpoint is defined at a single timepoint, and ANCOVA adjusting for the baseline value is the more efficient and less biased estimator. The 6- and 12-month outcomes, and all secondary outcomes, will be analyzed using analogous baseline-adjusted models and are considered secondary. Partial eta squared (η^2^) will be reported as a measure of effect size (small 0.0–0.13, medium 0.13–0.26, large > 0.26). All primary and secondary outcomes will be analyzed following the intention-to-treat (ITT) principle, which is prespecified as the primary analysis strategy: every randomized participant will be included in the group to which they were allocated, irrespective of adherence, protocol deviations, or discontinuation of the allocated intervention. This strategy is fixed a priori and is not contingent on the observed pattern of missing data. Missing data will be assumed to be missing at random and handled using multiple imputations by chained equations (m = 50 imputed datasets) [42]. The imputation model will include the baseline value of the outcome, treatment allocation, age, sex, body mass index, graft type, and all post-baseline outcome measurements, with estimates pooled using Rubin’s rules. The robustness of the findings will be examined in prespecified sensitivity analyses, subordinate to the primary ITT analysis, comprising a complete-case analysis, a per-protocol analysis (participants achieving ≥ 80% adherence), and a pattern-mixture (tipping-point) analysis to test the sensitivity of the results to plausible departures from the missing-at-random assumption [42]. Analyses will be conducted in SPSS Statistics version 26 (IBM Corp., Armonk, NY, USA) and GraphPad Prism version 10.0 (GraphPad Software, San Diego, CA, USA).

### 3.1. Harms

To collect, assess, report, and manage the potential adverse effects of the interventions that will be performed in this study, at the beginning and end of each treatment session, the participants from both groups will have a logbook available. In the same way, the collaborating orthopedic surgeons are members of the study team at the participating centers and provide cover; any participant reporting a clinically relevant increase in symptoms will be triaged by the treating physiotherapist and seen by a study surgeon promptly (as soon as possible and within 48–72 h). Finally, 1 year after surgery, each participant will be contacted by telephone to determine their condition and to evaluate whether they have experienced a re-tear of their ACL.

### 3.2. Ethics

This study will be conducted under the Declaration of Helsinki principles, as well as following the norms of Good Clinical Practice (GCP) [43]. This study protocol has been approved to the Scientific Ethical Committee of Clinica INDISA (ID: 019-2022) of the Metropolitan Region in Santiago, Chile. This research has been registered at the OSF registries with the Universal Trial Number (UTN) U1111-1288-0767 (https://doi.org/10.17605/OSF.IO/SRKHP). Any substantial protocol modification (e.g., to eligibility criteria, outcomes, or analyses) will be submitted to the Scientific Ethics Committee of Clínica INDISA for approval before implementation; approved amendments will be updated in the trial registry and reported in the final publication. Non-substantial administrative changes will be recorded in a dated protocol version log.

## 4. Discussion

This protocol describes the rationale and methods of a randomized clinical trial that will assess the effectiveness of adding a novel physiotherapy program to standard treatment to improve clinical outcomes in patients with ACL reconstruction. The intervention group will receive a standard physiotherapy program in addition to cross-education with visual biofeedback, mirror therapy, and virtual reality exercises. The control group will receive a standard physiotherapy program based on the consensus statement of the Sport and Health Rehabilitation Guidelines MOON Group.

A recent meta-analysis of transcranial magnetic stimulation studies of quadriceps showed significantly higher motor thresholds and motor-evoked potentials, and also significantly reduced short-interval intracortical inhibition compared with the uninjured side or healthy controls in patients after ACL injury and/or reconstruction, demonstrating brain changes in these patients [44]. Another meta-analysis reported that one in three patients may present with loss of extension of at least 3 degrees at the 12-month follow-up, decreasing to one in four patients at 2 years following ACL tear and/or reconstruction [41]. These findings are associated with arthrogenic muscle inhibition, a process where neural inhibition prevents adequate activation of the quadriceps muscle [45]. Its persistence can cause impaired maximal voluntary muscle activation, weakness, muscle atrophy and decreased ROM in ACL reconstruction, thus becoming a major barrier during the process of rehabilitation following ACL tear and/or reconstruction [45]. These impairments justify the need to evaluate new physiotherapy interventions.

Cross-education has shown positive results in improving function and quadriceps and hamstring strength after ACL reconstruction [46]. However, the results differ depending on the time of evaluation and the variables assessed. These discrepancies may be due to differences in the planning of therapeutic exercises and the variables used for assessments. On the other hand, due to the common neurophysiological mechanisms of neural plasticity in the cerebral cortex, and the cognitive–affective mechanisms between motor imagery, virtual reality, and cross-education, the effects of these therapies could be enhanced when they are combined [47,48]. Some studies on post-surgery patients have shown positive effects of motor imagery combined with standard rehabilitation on pain intensity, kinesiophobia, and muscle strength [49,50]. Conversely, a previous systematic review provided no clear evidence that motor imagery is an effective intervention after ACL reconstruction [51]. The inconsistency of the results could be due to differences between with patients with osteoarthritis and ACL injury. Regarding the mechanisms in these clinical conditions, the pathophysiological mechanisms involved are different, which may influence the outcomes and responses of patients to interventions in the early stages [50,51]. The evidence for virtual reality therapy is consistent. Based on meta-analyses, it can be said that virtual reality therapy alone has positive effects that are superior to traditional therapy on functionality, strength, pain intensity and joint range [52]. The beneficial effects of this therapy are based on the fact that it facilitates cognitive distraction and attentional engagement, and redirects focus away from the perception of pain and fear of movement [52,53]. In addition, virtual reality activates neural pathways that allow for optimizing neuromuscular coordination and improve the proprioception of the lower limb [53]. Beyond virtual reality, other technology-assisted approaches have also been used to support functional rehabilitation. For example, mechatronic and robotic-assisted gait training systems have been developed to provide controlled, repetitive, task-specific movement patterns that facilitate neuromuscular re-education and motor relearning; a usability pilot study of one such mechatronic gait rehabilitation system reported good acceptance among patients and clinicians, supporting the feasibility of incorporating device-assisted training into rehabilitation protocols [54,55]. These assisted rehabilitation strategies, together with virtual reality and cross-education, illustrate the growing trend toward multimodal, technology-supported rehabilitation programs aimed at optimizing motor recovery after musculoskeletal and neurological injuries, including ACL reconstruction.

The main strength of this protocol is its consideration of important methodological factors. The participants will be randomly assigned to both groups through a hidden allocation sequence. Furthermore, the sample size has been adjusted to consider the possibility of losses or dropouts, increasing the number of patients recruited by 20%, and, in the event of losses or withdrawals, a statistical analysis will be conducted by intention to treat. To minimize measurement bias, all evaluations will be performed by trained physiotherapists who are not part of the research team and will remain blinded in relation to the treatment groups. The statistician will also remain blinded to the group assignment. The outcome measures are suitable and frequently used in clinical practice, and they have a good level of validity and reliability. Finally, several further limitations should be acknowledged. First, it will not be possible to blind the participants and the physiotherapists involved in the study due to the nature of the interventions being studied. Second, self-report questionnaires will be used to assess some outcomes; they are prone to subjectivity and recollection bias. Third, the trial will be conducted at a single center, which may limit the generalizability of the findings. Eligibility is restricted to participants with a normal body mass index (18.50–24.99 kg/m^2^), so the results may not extend to overweight or obese patients. Fourth, because the experimental program combines three components (cross-education, mirror therapy, and virtual reality), the design does not allow the isolated effect of any individual component to be determined. Such an effect might reflect cross-education, mirror therapy, virtual reality, or their interaction, and could additionally be influenced by non-specific factors that co-vary with the experimental arm—greater therapist attention, the novelty of technology, higher patient engagement and motivation, and a larger effective therapeutic dose. Therefore, the trial cannot support component-specific causal inference, which is beyond its scope. Finally, the sample size was powered for the primary outcome; the trial may therefore be underpowered to detect between-group differences in the secondary and longer-term (12-month) outcomes, which should accordingly be interpreted as exploratory. In addition, the 12-month follow-up does not extend to formal return-to-sport testing; future studies should incorporate objective, criterion-based return-to-sport test batteries as recommended in contemporary consensus recommendations [56].

## 5. Conclusions

This protocol describes a randomized controlled trial designed to evaluate whether adding a multimodal intervention—cross-education with visual biofeedback, mirror therapy, and virtual reality—to a standard physiotherapy program provides an additional benefit over standard rehabilitation alone for passive knee extension and other clinical outcomes after ACL reconstruction. Because no outcome data are yet available, no conclusions can be drawn regarding clinical effectiveness. If the adjunctive multimodal intervention proves superior to standard rehabilitation, the findings may inform the development of future multimodal rehabilitation strategies for this population; if it does not, the results will nonetheless help clarify the role of these combined modalities and guide the design of subsequent trials.

## Data Availability

The datasets used and/or analyzed during the current study will be available from the corresponding author on reasonable request.

## Supplementary Materials

The following supporting information can be downloaded at https://www.mdpi.com/article/10.3390/jcm15145552/s1, SPIRIT 2013 Checklist: Recommended items to address in a clinical trial protocol and related documents.

## Abbreviations

The following abbreviations are used in this manuscript:

ACL	Anterior Cruciate Ligament
ANCOVA	Analysis of Covariance
BMI	Body Mass Index
eCRF	Electronic Care Report Form
GMI	Graded Motor Imagery
GCP	Good Clinical Practice
GPAQ	Global Physical Activity Questionnaire
ICC	Intraclass Correlation Coefficient
KOOS	Knee Injury and Osteoarthritis Outcome Score
MCID	Minimal Clinically Important Difference
PASS-20	Pain Anxiety Symptoms Scale Short Form 20
ROM	Range of Motion
SPIRIT	Standard Protocol Items: Recommendations for Interventional Trials
TENS	Transcutaneous Electrical Nerve Stimulation
TSK-17	Tampa Scale of Kinesiophobia (17-Item)
VAS	Visual Analog Scale
