# Supplementary material for: Effectiveness of the Addition of a Cross-Education, Mirror Therapy, and Virtual Reality Exercises in Patients with Anterior Cruciate Ligament Reconstruction: The CROSSMIRV Trial Protocol
Source: J Clin Med. 2026 Jul 15;15(14):5552. doi: 10.3390/jcm15145552 (PMC13412849; doi:10.3390/jcm15145552)
Supplement: Supplementary file 1 [file jcm-15-05552-s001.zip › jcm-4383388-supplementary.pdf]

SPIRIT
STANDARD PROTOCOL ITEMS: RECOMMENDATIONS FOR INTERVENTIONAL TRIALS

SPIRIT 2013 Checklist: Recommended items to address in a clinical trial protocol and related documents*

Trial: Effectiveness of cross-education, mirror therapy, and virtual reality added to standard physiotherapy in patients after ACL reconstruction — The CROSSMIRV Trial Protocol

Column key — Addressed in Manuscript: location and page number within Manuscript_original.docx. **Reviewer Comments & Recommendations:** item status (ADDRESSED / PARTIALLY ADDRESSED / NOT ADDRESSED / N/A) and specific guidance to improve the protocol before submission.

Section/item	Item No	Description	Addressed in Manuscript (Location / Page)	Reviewer Comments & Recommendations
**Administrative information**				
Title	1	Descriptive title identifying the study design, population, interventions, and, if applicable, trial acronym	Title page, p. 1	PARTIALLY ADDRESSED. Title includes the CROSSMIRV acronym and lists the three interventions. However, the study population (post-ACL reconstruction patients) and trial framework (superiority) are not explicit. Recommended revision: "CROSSMIRV trial: protocol for a single-blind, parallel-group superiority randomized controlled

Section/item	Item No	Description	Addressed in Manuscript (Location / Page)	Reviewer Comments & Recommendations
				trial evaluating cross-education, mirror therapy, and virtual reality in patients after ACL reconstruction."
Trial registration	2a	Trial identifier and registry name. If not yet registered, name of intended registry	Abstract, p. 1	PARTIALLY ADDRESSED. Registered at OSF Registries (UTN U1111-1288-0767; https://doi.org/10.17605/OSF.IO/SRKHP). The UTN number indicates the intent to register with WHO ICTRP; the corresponding primary registration should be confirmed and its identifier reported.
	2b	All items from the World Health Organization Trial Registration Data Set	Not reported	NOT ADDRESSED. The 24-item WHO Trial Registration Data Set has not been presented in the manuscript. These items

Section/item	Item No	Description	Addressed in Manuscript (Location / Page)	Reviewer Comments & Recommendations
				must be completed in the primary registry) and the registry identifier cited in the manuscript.
Protocol version	3	Date and version identifier	Title page, p. 1 (revised manuscript)	ADDRESSED. The following has been added to the title page: ‘Protocol version 1.0, dated [July/2026 ’ version and date will be recorded in the trial registry.
Funding	4	Sources and types of financial, material, and other support	Funding section, p. 15	ADDRESSED. The authors did not receive any financial support for the investigation, authorship, and/or publication of this article.
Roles and responsibilities	5a	Names, affiliations, and roles of protocol contributors	Title page, p. 1; Authors' contributions, p. 15	ADDRESSED. Six authors are listed with institutional affiliations. Individual contributions are described per ICMJE criteria in the Authors'

Section/item	Item No	Description	Addressed in Manuscript (Location / Page)	Reviewer Comments & Recommendations
				contributions section.
	5b	Name and contact information for the trial sponsor	Methods – new subsection 'Trial sponsorship and oversight', p. XX (revised)	ADDRESSED This is an investigator-initiated trial with no external sponsor. The principal investigator, FA-Q (Universidad San Sebastian, Santiago, Chile; felipe.arayaq@uss.cl. assumes the responsibilities of trial sponsor.'
	5c	Role of study sponsor and funders, if any, in study design; collection, management, analysis, and interpretation of data; writing of the report; and the decision to submit the report for publication, including whether they will have ultimate authority over any of these activities	Methods – 'Trial sponsorship and oversight', p. XX (revised)	ADDRESSED Added: 'As an investigator-initiated trial without an external sponsor or funder, all responsibility for study design; collection, management, analysis and interpretation of data; writing of the report; and the decision to submit for publication rests solely with the investigators.

Section/item	Item No	Description	Addressed in Manuscript (Location / Page)	Reviewer Comments & Recommendations
				No external sponsor or funder had any role in, or ultimate authority over, any of these activities.'
	5d	Composition, roles, and responsibilities of the coordinating centre, steering committee, endpoint adjudication committee, data management team, and other individuals or groups overseeing the trial, if applicable (see Item 21a for data monitoring committee)	Section 2.3, pp. 5–10	PARTIALLY ADDRESSED. Blinded outcome assessors and a blinded statistician are mentioned. No formal steering committee or coordinating centre is described. For a single-centre n=58 trial this is acceptable; add a brief paragraph describing the trial governance structure.
**Introduction**				
Background and rationale	6a	Description of research question and justification for undertaking the trial, including summary of relevant studies (published and unpublished) examining benefits and harms for each intervention	Section 1, pp. 1–3	ADDRESSED. The introduction provides the epidemiological burden of ACL injury, neurophysiological rationale for cross-education,

Section/item	Item No	Description	Addressed in Manuscript (Location / Page)	Reviewer Comments & Recommendations
				mirror therapy, and virtual reality, and a review of published evidence for each intervention. References 1–29 are pertinent. The absence of previous trials combining these three modalities is clearly stated.
	6b	Explanation for choice of comparators	Section 1, p. 3; Section 2.3, p. 5	PARTIALLY ADDRESSED. The MOON Group consensus protocol is used as the active comparator, and its clinical relevance is implied. However, no explicit sentence justifies why this specific protocol was chosen over alternatives. Add: "The MOON Group consensus protocol was selected as the comparator

Section/item	Item No	Description	Addressed in Manuscript (Location / Page)	Reviewer Comments & Recommendations
				because it represents the most widely disseminated evidence-based standard of care for post-ACL rehabilitation in Chile and internationally."
Objectives	7	Specific objectives or hypotheses	Section 1, p. 3	PARTIALLY ADDRESSED. The stated aim describes the objective of the protocol article ("to describe the rationale and methods"), not the trial hypothesis. A clear, testable hypothesis for the trial must be added: "We hypothesize that patients receiving the novel program (cross-education + mirror therapy + VR + standard physiotherapy) will achieve significantly greater improvement

Section/item	Item No	Description	Addressed in Manuscript (Location / Page)	Reviewer Comments & Recommendations
				in passive knee extension ROM at 6 weeks post-surgery compared to patients receiving standard physiotherapy alone."
**Trial design**				
Trial design	8	Description of trial design including type of trial (eg, parallel group, crossover, factorial, single group), allocation ratio, and framework (eg, superiority, equivalence, noninferiority, exploratory)	Section 2.1, p. 4	PARTIALLY ADDRESSED. Described as "single-blind, randomized controlled trial with two parallel groups" with 1:1 allocation. The trial framework (superiority) is not explicitly stated. Add: "This is a superiority trial. We hypothesize that the experimental intervention will produce clinically meaningful larger improvements than the control intervention."
**Methods: Participants, interventions, and outcomes**				

Section/item	Item No	Description	Addressed in Manuscript (Location / Page)	Reviewer Comments & Recommendations
Study setting	9	Description of study settings (eg, community clinic, academic hospital) and list of countries where data will be collected. Reference to where list of study sites can be obtained	Section 2.1, p. 4	ADDRESSED. Data will be collected at the Physical Therapy Department of the Chilean Medical Center, Santiago, Chile. Single-centre, single-country trial. Sufficient for a phase II protocol.
Eligibility criteria	10	Inclusion and exclusion criteria for participants. If applicable, eligibility criteria for study centres and individuals who will perform the interventions (eg, surgeons, psychotherapists)	Sections 2.2.1–2.2.2, pp. 4–5	ADDRESSED. Three inclusion criteria (age 18–50, ACL reconstruction, BMI 18.50–24.99, Spanish comprehension, Mini-Mental >26) and four exclusion criteria (inability to walk, comorbid knee pathology, orthotic devices, cognitive impairment) are clearly listed. Physiotherapists delivering interventions are required

Section/item	Item No	Description	Addressed in Manuscript (Location / Page)	Reviewer Comments & Recommendations
				to have >10 years of musculoskeletal experience; this eligibility criterion for interventionists could be made more explicit as a formal criterion.
Interventions	11a	Interventions for each group with sufficient detail to allow replication, including how and when they will be administered	Section 2.3, pp. 5–10	ADDRESSED. Both interventions (control: MOON Group protocol; experimental: MOON Group + cross-education with visual biofeedback + mirror therapy + VR) are described in detail by stage (0–6, 6–12, 12–24 weeks), including sets, repetitions, duration, equipment (Corpus VR InMotionVR / Oculus Meta Quest 3S), and session frequency (3×/week for 24 weeks). Figures 2 and

Section/item	Item No	Description	Addressed in Manuscript (Location / Page)	Reviewer Comments & Recommendations
				3 illustrate the novel components.
	11b	Criteria for discontinuing or modifying allocated interventions for a given trial participant (eg, drug dose change in response to harms, participant request, or improving/worsening disease)	Not reported	NOT ADDRESSED. No formal criteria for individual participant discontinuation are specified. Add: e.g., "Allocated intervention will be discontinued for a participant who: (1) experiences ACL re-rupture or major surgical complication; (2) develops pain consistently >7/10 VAS that does not resolve within 48 hours post-session; (3) withdraws consent; or (4) is unable to attend ≥2 consecutive scheduled sessions."
	11c	Strategies to improve adherence to intervention protocols, and any procedures for monitoring adherence (eg, drug tablet return, laboratory tests)	Section 2.3, p. 10	PARTIALLY ADDRESSED. Periodic reporting of clinical progression is mentioned

Section/item	Item No	Description	Addressed in Manuscript (Location / Page)	Reviewer Comments & Recommendations
				as a motivational strategy. Adherence will be recorded via eCRF session logs. A formal protocol for managing missed sessions (e.g., phone follow-up, rescheduling policy, adherence threshold for per-protocol analysis) is absent. Add a structured adherence monitoring plan.
	11d	Relevant concomitant care and interventions that are permitted or prohibited during the trial	Not reported	NOT ADDRESSED. Post-operative NSAIDs are mentioned (14-day course) but no explicit list of permitted or prohibited co-interventions is provided. Add: e.g., "Participants may receive prescribed analgesics and anti-inflammatory medications as directed

Section/item	Item No	Description	Addressed in Manuscript (Location / Page)	Reviewer Comments & Recommendations
				by their surgeon. Additional physical therapy outside the study protocol and corticosteroid injections are not permitted during the 24-week intervention period. Orthopaedic follow-up visits are encouraged."
Outcomes	12	Primary, secondary, and other outcomes, including the specific measurement variable (eg, systolic blood pressure), analysis metric (eg, change from baseline, final value, time to event), method of aggregation (eg, median, proportion), and time point for each outcome. Explanation of the clinical relevance of chosen efficacy and harm outcomes is strongly recommended	Section 2.4, pp. 8–10	PARTIALLY ADDRESSED. Primary outcome (passive knee extension ROM by goniometry) and five secondary outcomes (VAS pain, TSK-17 kinesiophobia, PASS-20 pain anxiety, KOOS knee function, isometric quadriceps strength, thigh perimeter) are described with instruments and psychometric properties.

Section/item	Item No	Description	Addressed in Manuscript (Location / Page)	Reviewer Comments & Recommendations
				Analysis metrics are only partially stated: the method of aggregation and specific time point comparisons are described in the statistical section but not in the outcomes section itself. For VAS and TSK-17, clinically accepted reference values exist in the literature and should be cited. The MCID of 18.3 for KOOS is correctly cited. The mechanistic rationale for choosing ROM as the primary outcome for neuromuscular interventions should be strengthened.
Participant timeline	13	Time schedule of enrolment, interventions (including any run-ins and washouts), assessments, and visits for participants. A schematic diagram is highly recommended (see Figure)	Figure 1; Sections 2.1, 2.4	ADDRESSED. Figure 1 presents the SPIRIT diagram with the schedule of enrolment, interventions,

Section/item	Item No	Description	Addressed in Manuscript (Location / Page)	Reviewer Comments & Recommendations
				and assessments. Four assessment time points are defined: baseline, 6 weeks, 24 weeks, and 12 months post-surgery. Verify that Figure 1 includes all secondary outcomes at each time point.
Sample size	14	Estimated number of participants needed to achieve study objectives and how it was determined, including clinical and statistical assumptions supporting any sample size calculations	Section 2.5, p. 10	PARTIALLY ADDRESSED. Sample size (n=58; 29/group) calculated with G*Power using Cohen's d=0.8, α=0.05, power=80%, +20% dropout adjustment. this trial). patients, (2) conduct a sensitivity analysis across a range of effect sizes, or (3) justify the assumption that d=0.8 is conservative for this intervention.

Section/item	Item No	Description	Addressed in Manuscript (Location / Page)	Reviewer Comments & Recommendations
Recruitment	15	Strategies for achieving adequate participant enrolment to reach target sample size	Section 2.6, p. 11	PARTIALLY ADDRESSED. Recruitment period (October 2026 – September 2027) is specified. The consent and information process is described. However, no active recruitment strategies are described (e.g., referral pathways from orthopaedic surgeons, clinic posters, screening logs). Add: recruitment sources, expected referral volume, and contingency plans if enrolment falls behind schedule.
**Methods: Assignment of interventions (for controlled trials)**				
Allocation:				
Sequence generation	16a	Method of generating the allocation sequence (eg, computer-generated random numbers), and list of any factors for stratification. To reduce predictability of a	Section 2.7, p. 11	PARTIALLY ADDRESSED. "A computer-generated sequence of

Section/item	Item No	Description	Addressed in Manuscript (Location / Page)	Reviewer Comments & Recommendations
		random sequence, details of any planned restriction (eg, blocking) should be provided in a separate document that is unavailable to those who enrol participants or assign interventions		numbers" is mentioned, but the specific software/tool, block size, and stratification factors are not provided. Add: e.g., "A computer-generated permuted-block randomization sequence (block size 4) will be generated using Research Randomizer (www.randomizer.org) by an investigator not involved in participant recruitment. No stratification factors are planned." Block size details should be kept in a sealed document separate from allocation.
Allocation concealment mechanism	16b	Mechanism of implementing the allocation sequence (eg, central telephone; sequentially numbered, opaque, sealed envelopes), describing any steps to conceal the sequence until interventions are assigned	Section 2.7, p. 11	ADDRESSED. Group assignments are kept in sealed envelopes accessible

Section/item	Item No	Description	Addressed in Manuscript (Location / Page)	Reviewer Comments & Recommendations
				only to the investigator managing allocation. This mechanism is appropriate for a single-centre phase II trial. Specify that envelopes are sequentially numbered and opaque.
Implementation	16c	Who will generate the allocation sequence, who will enrol participants, and who will assign participants to interventions	Section 2.7, p. 11	PARTIALLY ADDRESSED. The text implies that one investigator manages allocation, but the roles are not explicitly separated by name/position. Add: "The allocation sequence will be generated by [HG-E/researcher, independent of recruitment]. Participant enrolment and eligibility screening will be performed by [name/role: FA-Q]. Intervention assignment will be

Section/item	Item No	Description	Addressed in Manuscript (Location / Page)	Reviewer Comments & Recommendations
				communicated to the treating physiotherapist by [name/role] after the participant has signed informed consent."
Blinding (masking):				
Blinding (masking)	17a	Who will be blinded after assignment to interventions (eg, trial participants, care providers, outcome assessors, data analysts), and how	Section 2.7, p. 11	ADDRESSED. Blinding of participants and treating physiotherapists is not possible due to the nature of the interventions (clearly acknowledged). Outcome assessors (two physiotherapists external to the research team) and the statistician will be blinded. Add a description of the physical and procedural measures to maintain assessor blinding (e.g., separate data collection rooms, prohibition on

Section/item	Item No	Description	Addressed in Manuscript (Location / Page)	Reviewer Comments & Recommendations
				discussing group assignment during assessments).
	17b	If blinded, circumstances under which unblinding is permissible, and procedure for revealing a participant's allocated intervention during the trial	Not reported	NOT ADDRESSED (for assessors). Add: "Unblinding of outcome assessors will only be permissible in the event of a serious adverse event requiring clinical judgment regarding the treatment received. Any unblinding event will be documented and reported to the Ethics Committee."
**Methods: Data collection, management, and analysis**				
Data collection methods	18a	Plans for assessment and collection of outcome, baseline, and other trial data, including any related processes to promote data quality (eg, duplicate measurements, training of assessors) and a description of study instruments (eg, questionnaires, laboratory tests) along with their reliability and validity, if known. Reference to where data	Section 2.4, pp. 8–10	ADDRESSED. All instruments are described with reliability and validity references: Baseline goniometer (ICC 0.90–0.99), VAS, TSK-17, PASS-20, KOOS,

Section/item	Item No	Description	Addressed in Manuscript (Location / Page)	Reviewer Comments & Recommendations
		collection forms can be found, if not in the protocol		ActivForce dynamometer, measuring tape (GPAQ). Evaluations performed by two trained blinded physiotherapists external to the research team. Goniometric measurements taken in triplicate with mean used for analysis. This is adequate.
	18b	Plans to promote participant retention and complete follow-up, including list of any outcome data to be collected for participants who discontinue or deviate from intervention protocols	Not reported	NOT ADDRESSED. No plan is described for managing participants who withdraw from the intervention. Add: "Participants who withdraw from the allocated intervention will be asked to continue with outcome assessments at all scheduled time points, where possible (intention-to-treat data collection). The reason

Section/item	Item No	Description	Addressed in Manuscript (Location / Page)	Reviewer Comments & Recommendations
				for withdrawal will be recorded. Outcome data collected prior to withdrawal will be retained and included in the intention-to-treat analysis."
Data management	19	Plans for data entry, coding, security, and storage, including any related processes to promote data quality (eg, double data entry; range checks for data values). Reference to where details of data management procedures can be found, if not in the protocol	Section 2.8, p. 12	PARTIALLY ADDRESSED. Data collection on paper forms with subsequent entry into an Excel spreadsheet is described. Personal information will be replaced by unique identifiers. The following elements are absent and should be added: (1) use of a validated electronic data capture system (REDCap is recommended and freely available); (2) double data entry or automated

Section/item	Item No	Description	Addressed in Manuscript (Location / Page)	Reviewer Comments & Recommendations
				range checks; (3) designation of a data custodian; (4) data storage location, security measures, and minimum retention period (recommended: 10 years after study completion); (5) data destruction procedures at end of retention period.
Statistical methods	20a	Statistical methods for analysing primary and secondary outcomes. Reference to where other details of the statistical analysis plan can be found, if not in the protocol	Section 2.9, pp. 12–13	ADDRESSED. Repeated measures ANCOVA adjusted for baseline values is specified as the primary analysis. Normality assessed by Kolmogorov-Smirnov test and Q-Q plots. Student's t-test for continuous baseline comparisons; chi-square for categorical variables. Partial eta squared (η^2) as effect size

Section/item	Item No	Description	Addressed in Manuscript (Location / Page)	Reviewer Comments & Recommendations
				measure. 95% CI reported. Software: SPSS v26 and GraphPad Prism v10. This is adequate.
	20b	Methods for any additional analyses (eg, subgroup and adjusted analyses)	Section 2.9, p. 12	PARTIALLY ADDRESSED. Covariate adjustment (baseline values of each outcome) is specified. No pre-specified subgroup analyses are described. If no subgroup analyses are planned, explicitly state: "No subgroup analyses are pre-specified. Any post-hoc exploratory analyses will be clearly labelled as such in the results."
	20c	Definition of analysis population relating to protocol non-adherence (eg, as randomised analysis), and any statistical methods to handle missing data (eg, multiple imputation)	Section 2.9, p. 13	PARTIALLY ADDRESSED. Intention-to-treat analysis is mentioned but stated to be "decided prior to the beginning of

Section/item	Item No	Description	Addressed in Manuscript (Location / Page)	Reviewer Comments & Recommendations
				the study"—this commitment must be explicit in the protocol itself. Missing data handling via multiple imputation using chained equations is mentioned. Add the following pre-commitment: "All randomised participants will be analysed in the group to which they were allocated (intention-to-treat principle). Multiple imputation by chained equations will be used for missing outcome data, with auxiliary variables included to satisfy the missing-at-random assumption."
**Methods: Monitoring**				
Data monitoring	21a	Composition of data monitoring committee (DMC); summary of its role and reporting structure;	Methods – Monitoring, new	ADDRESSED (revised protocol).

Section/item	Item No	Description	Addressed in Manuscript (Location / Page)	Reviewer Comments & Recommendations
		statement of whether it is independent from the sponsor and competing interests; and reference to where further details about its charter can be found, if not in the protocol. Alternatively, an explanation of why a DMC is not needed	subsection, p. XX (revised)	Justification added: ‘Given the low-risk, exercise-based nature of the interventions, the small single-centre sample (n=58), and the absence of any planned interim efficacy analyses, a formal independent Data Monitoring Committee was not established. Safety oversight is provided by the principal investigator (FA-Q), who reviews adverse-event logs and adherence rates after every 10 participants complete the 6-week primary-outcome assessment and may consult the Scientific Ethics Committee of Clínica INDISA if any

Section/item	Item No	Description	Addressed in Manuscript (Location / Page)	Reviewer Comments & Recommendations
				safety concern arises.’
	21b	Description of any interim analyses and stopping guidelines, including who will have access to these interim results and make the final decision to terminate the trial	Methods – Monitoring, p. XX (revised)	ADDRESSED No interim efficacy analyses are planned and the trial will not be stopped early for efficacy. The principal investigator retains authority to suspend or terminate the trial for safety reasons – for example, if trial-related serious adverse events occur – in consultation with the Scientific Ethics Committee of Clínica INDISA.’
Harms	22	Plans for collecting, assessing, reporting, and managing solicited and spontaneously reported adverse events and other unintended effects of trial interventions or trial conduct	Section 2.10, p. 13	ADDRESSED. Adverse events are recorded in a logbook at the start and end of each session. Participants with symptom exacerbation within 48 hours will be referred immediately

Section/item	Item No	Description	Addressed in Manuscript (Location / Page)	Reviewer Comments & Recommendations
				to an orthopaedic surgeon. Telephone follow-up at 12 months to assess ACL re-rupture. Strengthen by: (1) providing a formal definition of adverse event and serious adverse event in this context; (2) specifying reporting timelines to the Ethics Committee for serious adverse events.
Auditing	23	Frequency and procedures for auditing trial conduct, if any, and whether the process will be independent from investigators and the sponsor	Methods – Monitoring, p. XX (revised)	ADDRESSED (revised protocol). Added: 'No independent external audit is planned. The principal investigator performs monthly internal monitoring of case-report-form completeness, adherence logs and adverse-event records to verify protocol

Section/item	Item No	Description	Addressed in Manuscript (Location / Page)	Reviewer Comments & Recommendations
				compliance; findings are documented and retained. As this is an investigator-initiated trial with no external sponsor, this monitoring is internal to the investigator team.’
**Ethics and dissemination**				
Research ethics approval	24	Plans for seeking research ethics committee/institutional review board (REC/IRB) approval	Section 2.11, p. 13; Ethics section, p. 14	ADDRESSED. Approved by the Scientific Ethics Committee of Clínica INDISA (ID: 019-2022) on December 5, 2022. Adherence to the Declaration of Helsinki and Good Clinical Practice guidelines is declared.
Protocol amendments	25	Plans for communicating important protocol modifications (eg, changes to eligibility criteria, outcomes, analyses) to relevant parties (eg, investigators, REC/IRBs, trial participants, trial registries, journals, regulators)	Methods, p. XX (revised)	ADDRESSED (revised protocol). Added: ‘Any substantial protocol modification (e.g. to eligibility criteria, outcomes or analyses) will

Section/item	Item No	Description	Addressed in Manuscript (Location / Page)	Reviewer Comments & Recommendations
				be submitted to the Scientific Ethics Committee of Clínica INDISA for approval before implementation; approved amendments will be updated in the trial registry and reported in the final publication. Non-substantial administrative changes will be recorded in a dated protocol version log.’
Consent or assent	26a	Who will obtain informed consent or assent from potential trial participants or authorised surrogates, and how (see Item 32)	Section 2.6, p. 11	ADDRESSED. The principal investigator (FA-Q) will provide verbal information and obtain written informed consent before enrolment. Participants may withdraw at any time without penalty. The consent form covers study

Section/item	Item No	Description	Addressed in Manuscript (Location / Page)	Reviewer Comments & Recommendations
				background, procedures, expected benefits, and potential risks.
	26b	Additional consent provisions for collection and use of participant data and biological specimens in ancillary studies, if applicable	N/A	NOT APPLICABLE. No biological specimens are collected and no ancillary studies are planned.
Confidentiality	27	How personal information about potential and enrolled participants will be collected, shared, and maintained in order to protect confidentiality before, during, and after the trial	Section 2.8, p. 12	PARTIALLY ADDRESSED. Participant names will be replaced by unique identifier numbers; data will be stored confidentially on a password-protected computer; compliance with Chile's Personal Data Protection Act (Law 19.628) is mentioned. Add: (1) who holds the decoding key linking identifiers to names; (2) whether data will be shared with third

Section/item	Item No	Description	Addressed in Manuscript (Location / Page)	Reviewer Comments & Recommendations
				parties and under what conditions; (3) data storage location, duration, and destruction procedures at end of retention period.
Declaration of interests	28	Financial and other competing interests for principal investigators for the overall trial and each study site	Competing interests, p. 15	ADDRESSED. All authors declare no competing interests: "The authors declare that they have no competing interests." Sufficient.
Access to data	29	Statement of who will have access to the final trial dataset, and disclosure of contractual agreements that limit such access for investigators	Availability of data, p. 15 (revised)	ADDRESSED ‘The final de-identified trial dataset will be accessible to the principal investigator (FA-Q) and the trial statistician. No contractual agreements limit investigator access to the data. De-identified participant-level data and the statistical analysis code will be made

Section/item	Item No	Description	Addressed in Manuscript (Location / Page)	Reviewer Comments & Recommendations
				available on reasonable request following publication.’
Ancillary and post-trial care	30	Provisions, if any, for ancillary and post-trial care, and for compensation to those who suffer harm from trial participation	Not reported	NOT ADDRESSED. No provision for post-trial care or compensation for trial-related harm is described. Add: "Participants who experience adverse events directly related to trial participation will be referred to the orthopaedic surgeon at Clínica INDISA at no additional cost to the participant. No financial compensation is provided for participation. Insurance coverage follows standard institutional policy."
Dissemination policy	31a	Plans for investigators and sponsor to communicate trial results to participants,	Discussion – new dissemination	ADDRESSED (revised protocol).

Section/item	Item No	Description	Addressed in Manuscript (Location / Page)	Reviewer Comments & Recommendations
		healthcare professionals, the public, and other relevant groups (eg, via publication, reporting in results databases, or other data sharing arrangements), including any publication restrictions	paragraph, p. XX (revised)	Trial results will be disseminated through open-access peer-reviewed publication regardless of the direction of the findings, presented at national and international rehabilitation conferences, and reported in the trial registry within 12 months of study completion. Participants who request it will be informed of the aggregate results. No publication restrictions apply.’
	31b	Authorship eligibility guidelines and any intended use of professional writers	Authors' contributions, p. 15	ADDRESSED. Individual author contributions per ICMJE criteria are listed. No professional writers are involved.
	31c	Plans, if any, for granting public access to the full protocol, participant-level dataset, and statistical code	Availability of data, p. 15	PARTIALLY ADDRESSED. Data sharing on request is

Section/item	Item No	Description	Addressed in Manuscript (Location / Page)	Reviewer Comments & Recommendations
				stated. Commit to: (1) depositing the full protocol (current version) in a public repository (e.g., OSF) before enrolment begins; (2) sharing de-identified participant-level data and statistical analysis code (SPSS syntax) upon reasonable request after publication.
**Appendices**				
Informed consent materials	32	Model consent form and other related documentation given to participants and authorised surrogates	Supplementary Material – Appendix S1	ADDRESSED (revised protocol). The model informed consent form provided to participants has been added as Supplementary Material (Appendix S1).
Biological specimens	33	Plans for collection, laboratory evaluation, and storage of biological specimens for genetic or molecular analysis in the current trial and for future use in ancillary studies, if applicable	N/A	NOT APPLICABLE. No biological specimens are collected in this trial.

Section/item	Item No	Description	Addressed in Manuscript (Location / Page)	Reviewer Comments & Recommendations
***** It is strongly recommended that this checklist be read in conjunction with the SPIRIT 2013 Explanation & Elaboration for important clarification on the items. Amendments to the protocol should be tracked and dated. The SPIRIT checklist is copyrighted by the SPIRIT Group under the Creative Commons "Attribution-NonCommercial-NoDerivs 3.0 Unported" license.				
